# Further Investigations Concerning Streptococci1Read at the Society for Internal Medicine, Berlin, March 23, 1903.

**Published:** 1903-07

**Authors:** Hans Aronson


### ABSTRACTS.

### Further Investigations Concerning Streptococci.[1]

By DR. HANS ARONSON.

Former experiments led the author (*Münchener Med. Wochenschrift*, April 7, 1903,) to the conclusion that there are no specific differences between the streptococci found in various infections, such as erysipelas, scarlet fever, articular rheumatism and sepsis.

1. Read at the Society for Internal Medicine, Berlin, March 23, 1903.

All streptococci may cause joint and heart affections in horses. The serum of a horse immunized with a certain variety of streptococci protects against and agglutinates in the same manner all streptococci.

In his more recent experiments, Aronson employed twenty different varieties of the streptococcus. They were administered to horses without previous animal passages, and their agglutinative powers were tested and compared with the immunising serum and that obtained from other streptococci. The agglutination must be macroscopically visible to be convincing. The experiments showed that horse serum markedly agglutinates only the material with which it has been treated; it does so little or not at all with other streptococci, even when they come from the same human disease. Agglutination is, therefore, an individual reaction. The specific nature of the scarlatina streptococcus cannot be demonstrated; therefore there cannot be a specific scarlatina serum.

A long series of experiments on mice, rabbits and dogs, proved that it is impracticable to prepare a serum from streptococcus cultures directly derived from human beings. He therefore adds to his serum, which is equably directed against all streptococci, and is obtained by treating a horse with highly virulent streptococci, a fluid produced by immunisation with streptococci taken directly from human subjects. Exact measurement of the latter has not yet been possible. A uniform action can only be secured by mixing the sera of different horses and by the use of numerous races.

In discussing this paper, Prof. A. Baginsky stated he had used Aronson's serum in 56 further cases of scarlet fever since his report to the Berlin Medical Society. The epidemic was a very severe one, for many of the other cases admitted at the same time into the hospital had bad sequellæ (glandular abscesses, hemorrhagic nephritis, and the like). He had gotten the distinct impression that many cases which were severe in the beginning, ran their course without complication by reason of the influence of the serum. The Aronson serum is at all events entirely innocuous; in suitable cases it fully deserves a trial in doses of 20 to 50 c.c.

